# Task-dependent plasticity in distributed neural circuits after transcranial direct current stimulation of the human motor cortex: A proof-of-concept study

**DOI:** 10.3389/fpain.2022.1005634

**Published:** 2022-11-25

**Authors:** Duncan J. Hodkinson, Stephen R. Jackson, JeYoung Jung

**Affiliations:** ^1^ Division of Clinical Neuroscience, School of Medicine, University of Nottingham, Nottingham, United Kingdom; ^2^ Sir Peter Mansfield Imaging Centre, School of Medicine, University of Nottingham, Nottingham, United Kingdom; ^3^ National Institute for Health Research (NIHR), Nottingham Biomedical Research Centre, Queens Medical Center, Nottingham, United Kingdom; ^4^ Versus Arthritis Pain Centre, University of Nottingham, Nottingham, United Kingdom; ^5^ School of Psychology, University of Nottingham, Nottingham, United Kingdom

**Keywords:** primary motor cortex, motor, tDCS, plasticity, connectivity, brain networks, fMRI

## Abstract

The ability of non-invasive brain stimulation to induce neuroplasticity and cause long-lasting functional changes is of considerable interest for the reversal of chronic pain and disability. Stimulation of the primary motor cortex (M1) has provided some of the most encouraging after-effects for therapeutic purposes, but little is known about its underlying mechanisms. In this study we combined transcranial Direct Current Stimulation (tDCS) and fMRI to measure changes in task-specific activity and interregional functional connectivity between M1 and the whole brain. Using a randomized counterbalanced sham-controlled design, we applied anodal and cathodal tDCS stimulation over the left M1. In agreement with previous studies, we demonstrate that tDCS applied to the target region induces task-specific facilitation of local brain activity after anodal tDCS, with the stimulation effects having a negative relationship to the resting motor threshold. Beyond the local effects, tDCS also induced changes in multiple downstream regions distinct from the motor system that may be important for therapeutic efficacy, including the operculo-insular and cingulate cortex. These results offer opportunities to improve outcomes of tDCS for the individual patient based on the degree of presumed neuroplasticity. Further research is still warranted to address the optimal stimulation targets and parameters for those with disease-specific symptoms of chronic pain.

## Introduction

In recent years there has been a move towards utilizing non-invasive brain stimulation (NIBS) and neuromodulation in the management of disability and chronic neurological diseases (1, 2). Of these, transcranial Direct Current Stimulation (tDCS) is a promising technique as it can change brain activity in a safe and reversable manner (3, 4). tDCS can be used independently or concomitantly with other treatments, thus offering more flexible therapeutic control to patients and physicians (5).

Evidence-based guidelines on the therapeutic use of tDCS in certain types of pain disorders have been characterized by stimulation of the primary motor cortex (M1) (6, 7). Additionally, M1-tDCS has shown *promise for motor rehabilitation therapy* (8)*.* Alongside these clinical studies, detailed experimental work has been performed to assess the safety and mechanisms underlying the observed effects of M1 stimulation under a wide range of conditions (9, 10). Current available evidence suggests that the acute effects of tDCS are driven by a shift in resting neuronal membrane potential, which depends on the polarity of the electric current relative to the orientation of cortical columns, while the after-effects involve synaptic plasticity (11–13). Studies exploring the polarity-specific effects of tDCS suggest that anodal stimulation can increase cortical excitability, while cathodal stimulation leads to a decrease in excitability within the cortex (14, 15).

Despite efforts to characterize tDCS-induced effects at the neuronal level, the underlying mechanisms driving the induction and expression of cortical plasticity in humans remains largely unknown. Experimental work suggests that tDCS can interact with various neurotransmitters in the brain (16–21), and may trigger changes in brain-derived neurotrophic factor (BDNF) (22). Beyond the local effects, tDCS can alter neuronal network physiology with significant effects on brain activity in remote interconnected cortical and subcortical areas (23–26). It has been proposed that such modifications in connectivity and synchronization are the result of changes in the fidelity of signal transmission by neurons (27). However, it is still not clear to what extent M1 stimulation modulates brain activity beyond the motor system.

In order to investigate the effects on neural networks, brain stimulation has been combined with neuroimaging techniques (28, 29). Using fMRI, widespread changes in functional activity and connectivity have been reported as a result of M1-tDCS. This includes evidence of modified coupling within motor networks due to cathodal and anodal stimulation (21, 30, 31), and reduced long-range connectivity due to anodal stimulation (25, 32, 33). Further studies combining MRS and fMRI approaches have found an inverse relationship between network strength and local inhibitory tone within M1 (21, 34, 35). These results clearly demonstrate that local application of tDCS can have wide-ranging neurobiological effects. However, currently the use of tDCS to modulate pain is limited by an incomplete understanding of the exact mechanisms by which tDCS affects brain function.

In the present study, we aim to establish the feasibility and rationale for use of tDCS to modulate clinically relevant brain systems beyond the motor network suitable for targeting pain. We combined tDCS and fMRI to measure changes in task-specific activity and interregional functional connectivity between the primary motor cortex (M1) and the whole brain. In a randomized counterbalanced sham-controlled design, we applied anodal tDCS (atDCS) and cathodal tDCS (ctDCS) stimulation over the left M1. We chose a well-established electrode montage, which has been reliability shown to modulate plasticity in both experimental and clinical settings (36). The purpose was to functionally characterize the after-effects of tDCS and determine the spatial extent of changes in neural activity following motor cortex stimulation.

## Materials and methods

### Participants

Twenty-seven healthy volunteers participated in this study (mean age, 23.3 ± 2.5 years; age range: 19–30 years, 10 males). Subject were randomized according to three stimulus types: anodal (*N* = 9, 5 males), cathodal (*N* = 9, 2 males), and sham (*N* = 9, 3 males). All participants were right handed assessed by the Edinburgh Handedness Inventory (37). We obtained written informed consent from all participants prior to the experiment. The work was approved by local research ethics committees at the University of Nottingham.

### Experimental design and procedures

**Figure 1:**
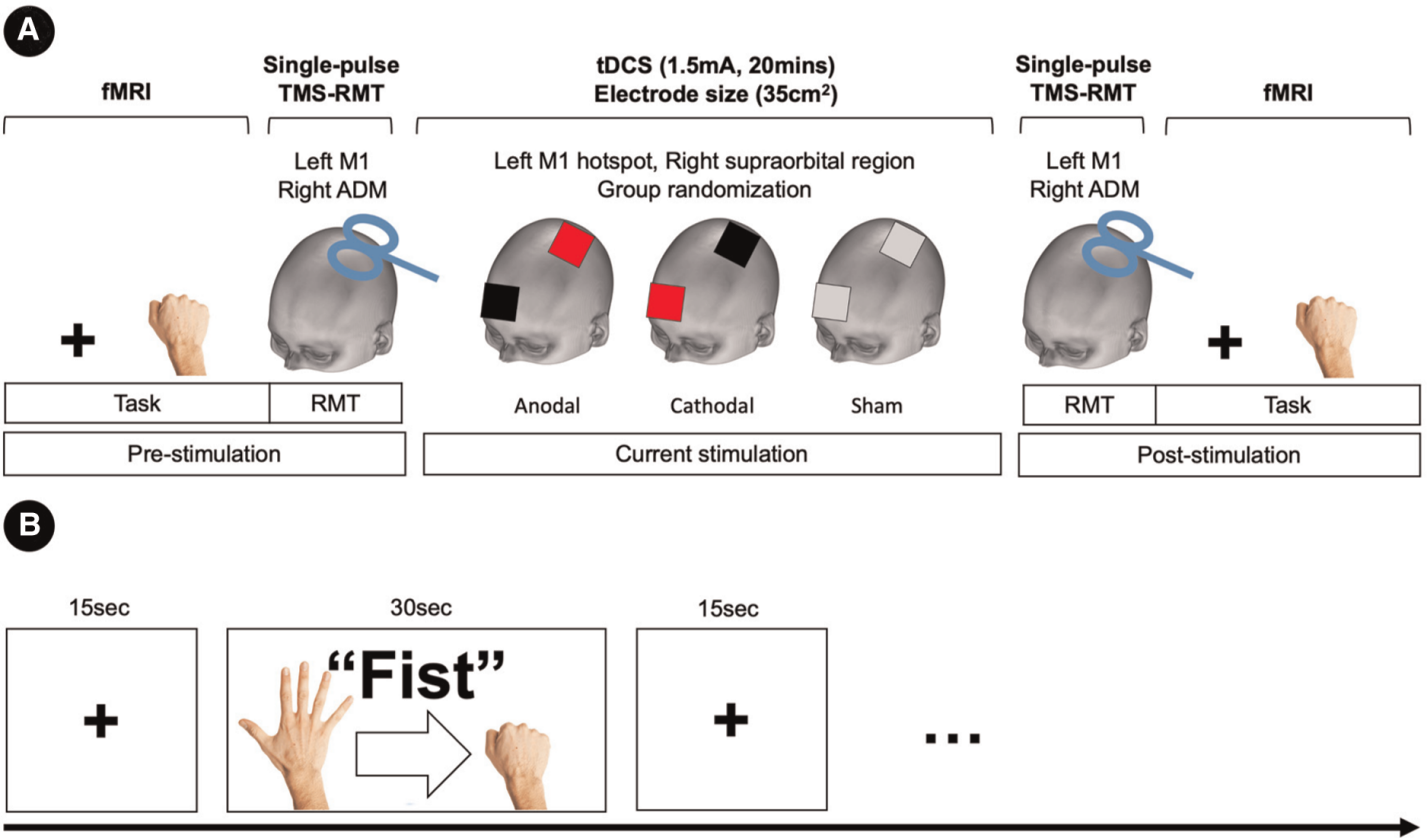
Experimental deign and procedures. (**A**) Task-fMRI and resting motor thresholds (RMTs) were assessed before and after tDCS stimulation (pre/post-stimulation). RMTs were elicited in the left primary motor cortex (M1) with single-pulse TMS of the abductor digiti minimi (ADM) muscle of the right hand. Subjects were assigned to a stimulus type in a randomized, counterbalanced design. Anodal stimulation is represented in red, cathodal stimulation in black, and sham in grey. (**B**) Block fMRI design for the hand motor task.

Subjects were assigned to the stimulus type of tDCS in a randomized, counterbalanced design. A between subject design was used to ensure all subjects were naïve to the tDCS stimulation. fMRI and resting motor thresholds (RMTs) were assessed before and after tDCS stimulation (pre/post-stimulation) (Figure 1A). The fMRI session consisted of resting-state and hand motor task. In the task condition, participants performed a simple hand clenching task. The hand clenching task required participant to clench and unclench continuously with their right hand when they saw the word “*Fist*” on the screen. We used an fMRI block design (total 10mins) consisting of a fist condition (30s) and a fixation (15s) (Figure 1B).

### Magnetic resonance imaging

Functional MR images were acquired at the Sir Peter Mansfield Imaging Centre, University of Nottingham, using a Philips Achieva 3.0-Tesla scanner equipped with an eight-channel SENSE head coil. Structural images were acquired using 3D MPRAGE sequence (1 mm^3^ isotropic resolution, repetition time (TR)/echo time (TE) = 8.278/2.3 ms, flip angle = 8°, 160 slices, FOV = 256 mm × 256 mm). For functional images, echo-planar imaging (EPI) sequence (TR/TE = 2,000/35 ms, flip angle 90°, 32 slices, FOV = 240 mm × 240 mm, voxel size = 3 mm × 3 mm × 3 mm) was used for the task and resting conditions.

### Transcranial direct current stimulation

Transcranial direct current stimulation (tDCS) was delivered through a pair of sponge electrodes (7 cm × 5 cm, 35 cm^2^). The electrodes were connected to a battery-driven constant-current stimulator (NeuroConn, Germany). In the experiment, the target electrode (anodal and cathodal) was placed over the hand motor hotspot within the left primary motor cortex (the right hand area) as identified by transcranial magnetic stimulation (TMS), and the other electrode was positioned over the right supraorbital region as a reference electrode. Current strength was 1.5 mA and the stimulation was delivered for 20 min (10 s for ramp up and down). For sham stimulation, the current (1.5 mA) was ramped up over 10 s then immediately turned off. tDCS was delivered the outside of the scanner, and there was a 10 min period between the tDCS and fMRI sessions (pre- and post-stimulation).

### Resting motor thresholds

To assess the tDCS-induced changes of an individual's RMT, we used TMS before and after the current stimulation. The TMS machine (Magstim Rapid2 stimulator, UK) was delivered using a figure-of-eight coil (70 mm outer wing diameter) and applied over the hand motor hotspot in the left M1. The hand motor hotspot was determined as the site elicited a muscle twitch of the right hand. RMTs were elicited in the left primary motor cortex (M1) with single-pulse TMS of the abductor digiti minimi (ADM) muscle of the right hand. The TMS coil was oriented perpendicular to the central sulcus at a 45° angle from the mid-sagittal line approximately. RMT was defined as the minimum stimulator output that induces an observable muscle twitch at the right hand for five of ten TMS pulses. We repeated the RMT three times and used the average value as a measure of corticospinal excitability.

### fMRI data analysis

Functional images were preprocessed and analysed with the Statistical Parametric Mapping software package (SPM12, Wellcome Department of Imaging Neuroscience, UK, http://www.fil.ion.ucl.ac.uk/spm/software/spm12/). Preprocessing included realignment, coregistration, normalization to the Montreal Neurological Institute (MNI) space, and spatial smoothing using a Gaussian kernel 8 mm full width half maximum (FWHM). For the task fMRI, a general linear model (GLM) was used to compute individual contrasts and a design matrix was comprised of the task condition and fMRI sessions (pre and post) at an individual level. Head movement parameters were included as regressors. For each participant, two contrasts were estimated (pre: fist > fixation and post: fist > fixation). In the random effect analysis, two-factorial ANOVA with the stimulation (anodal, cathodal, and sham) and session (pre and post) was performed. Statistical significance was set at *p* < 0.005 at a voxel level and *p* < 0.05, ks > 30 at a cluster level with false-discovery rate (FDR) correction.

### Functional connectivity analysis

We used CONN toolbox (CONN 20.b, http://web.mit.edu/swg/software.html) to estimate functional connectivity (FC) from a seed area to the whole brain. Prepocessed images were entered into the toolbox. Denoising using the component-based noise correction method (CompCor) implemented in the CONN toolbox (38) was conducted to remove white matter, cerebrospinal fluid, and physiological noise as well as head motion related artifacts. The data was also detrended, despiked, and filtered with a band pass (0.001 < *f* <0.008 Hz) to remove slow fluctuating signal such as scanner drift. Head movements were taken into account and motion parameters and their first-order temporal derivatives were regressed out. The onset and duration of the experimental condition (fist and fixation) were entered to the toolbox to generate FC for each condition in the fMRI task.

### Generalized psychophysiological interaction analysis

In order to examine the interaction effects of tDCS from the left M1 to other brain regions, we used a generalised psychophysiological interaction (gPPI) analysis implemented in CONN toolbox (39). PPI analysis can be used to identify regions that differ in connectivity by context or condition in fMRI studies, which makes inferences of context-dependent functional integration between a seed and other regions (40). Compared to the standard PPI analysis, the gPPI reduces both false negatives and false positives, especially in experiments involving more than two conditions (39, 41).

To perform the gPPI, we extracted an averaged blood oxygenation level-dependent (BOLD) time-course from a seed region as the physiological regressor. We generated a PPI regressor for each condition as the product between the psychological (the vector of the condition) and physiological regressors. The physiological and PPI regressors were convolved with the hemodynamic response function. We compared voxel-wise how strongly the time-course of a seed/ROI is correlated with the PPI regressors of another. At a group-level, we performed a random-effects analysis to measure differences in FC between the active stimulation (anodal and cathodal) and sham stimulation after the stimulation. For the seed to whole brain connectivity, statistical significance was set at *p* < 0.01 at the voxel level with FDR-corrected cluster threshold of *p* < 0.05, ks > 30 (42).

## Results

### RMT results

**Figure 2:**
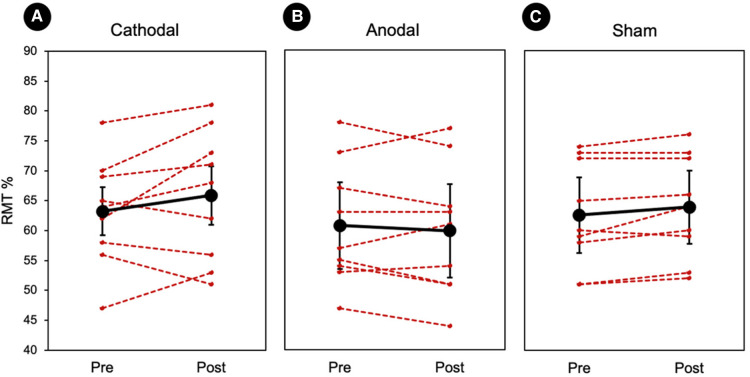
Individual resting motor thresholds. (**A–C**) Three randomized tDCS groups. Red represents each individual and the black represents the group average. There was no significant difference between the session (pre vs. post) or groups (cathodal vs. anodal vs. sham). Error bars represents standard errors.

A planned comparison of RMTs was conducted within each stimulation group. Cathodal stimulation showed a minor increase in RMT after stimulation (pre: 63.12% ± 3.06, post: 65.74 ± 3.72), however this effect was not significant. Anodal stimulation (pre: 61.07% ± 3.59, post: 60.00 ± 3.60) and sham (pre: 62.48% ± 2.91, post: 63.55 ± 2.76) also did not show any significant changes in the RMT. At the group-level, there was no significant effect of stimulus type (*Z* < −1.12, ps > 0.15). The results of the RMT analysis and individual values are displayed in Figure 2.

### Whole-brain GLM results

**Figure 3:**
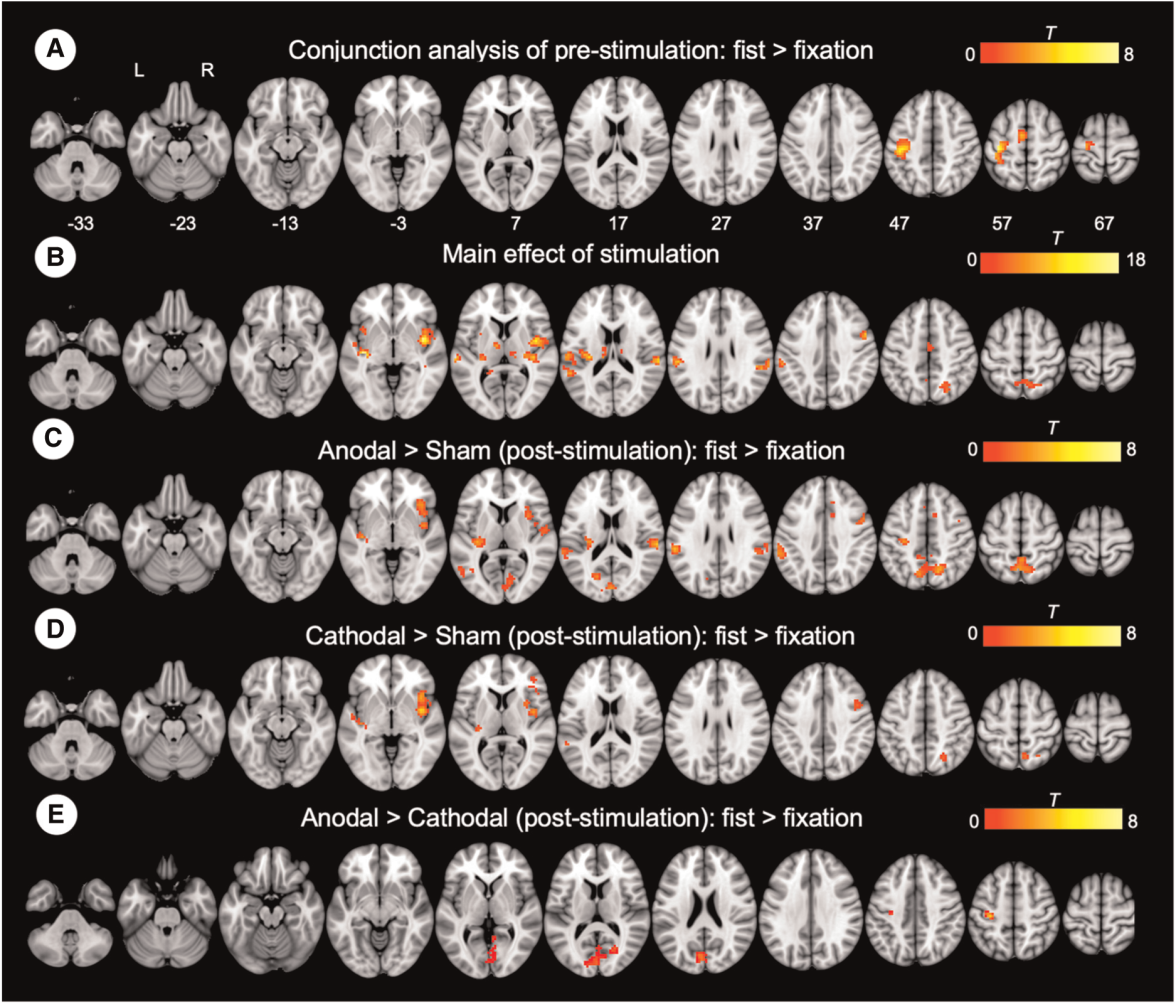
Whole-brain GLM results. (**A**) Conjunction analysis for the pre-stimulation task response across the groups. (**B**) Main effect of stimulation. (**C**) Comparison between anodal and sham group. (**D**) Comparison between cathodal and sham group. (**E**) Comparison between anodal and cathodal group. R (right) and L (left) indicate image orientations for slices in axial planes. Reference positions are in MNI coordinates. All images are displayed with a statistical significance of *p* < 0.005 at the voxel level and *p* < 0.05 (ks > 30) at a cluster level with false-discovery rate (FDR) correction.

To examine the effect of task (fist > fixation) prior to the stimulation, we performed a conjunction analysis across the stimulation groups (anodal, cathodal, and sham). The hand motor task induced significant activations in the left M1 and supplementary motor cortex (Figure 3A). In addition, the main effect of stimulation revealed significant activations in brain areas, including the bilateral insula, operculum, middle cingulate cortex, thalamus, and precuneus (Figure 3B). To examine the polarity-specific effects, we compared the active tDCS groups with the sham group in the post-stimulation session. The anodal group showed increased activation in the insula, operculum, thalamus, and cuneus (Figure 3C). The cathodal group revealed a similar pattern of activity, but only the right insula showed a significant activation compared to sham (Figure 3D). In comparison of active stimulation groups, the anodal group showed increased activation in the visual cortex, whereas the cathodal group did not show any significant activation (Figure 3E). There were no significant effects in the comparisons between pre- and post-stimulation for all groups. In addition, there were no significant effects pre-stimulation between the groups. These results are summarized in Table 1.

**Table 1:** Results of the whole-brain GLM analysis.

Contrast	Regions	Cluster size	*Z*	MNI coordinate		
				x	y	z
Conjunction analysis						
Pre-session	Precentral gyrus	237	5.61	−39	−24	54
fist > fixation			2.76	−15	−18	66
	Supplementary motor area	66	4.23	−6	−9	54
Main effect of stimulation						
	Insula	261	4.57	39	−3	3
			3.54	39	−24	6
			3.4	30	−24	9
	Insula	135	4.21	−39	−18	−3
			3.9	−27	−27	12
	Central operculum		3.36	−36	−21	18
	Parietal operculum	94	3.82	60	−30	21
			3.58	51	−36	24
	Middle temporal gyrus		3.22	57	−45	12
			3.77	−12	−36	12
	Thalamus	104	3.37	−9	−21	15
			3.28	−9	−9	6
	Central operculum	187	3.74	−60	−24	12
	Parietal operculum		3.54	−54	−30	27
	Superior temporal gyrus		3.53	−57	−45	15
	Middle cingulate cortex	74	3.46	9	−21	42
			3.09	3	−12	45
	Superior Parietal lobe	122	3.44	24	−66	42
			3.31	15	−63	51
	Precuneus		2.95	0	−54	54
Anodal > Sham						
Post-sessoion	Thalamus	152	4.5	−27	−27	12
fist > fixation	Parietal operculum	105	4.1	60	−27	18
	Precuneus	275	4.06	12	−63	51
			3.97	−3	−63	54
			3.46	3	−48	57
	Insula	108	3.86	36	24	−6
			3.55	33	9	9
			3.17	42	12	−3
	Cuneus	160	3.64	−18	−72	21
			3.6	0	−84	15
	Calcarine cortex		3.13	9	−75	9
Cathodal > Sham						
Post-sessoion	Insula	214	4.61	39	0	0
fist > fixation			3.73	39	15	−3
			3.38	33	6	3
Anodal > Cathodal						
Post-sessoion	Calcarine cortex	243	3.51	−12	−87	15
fist > fixation	Cuneus		3.46	0	−84	18

### ROI results

**Figure 4:**
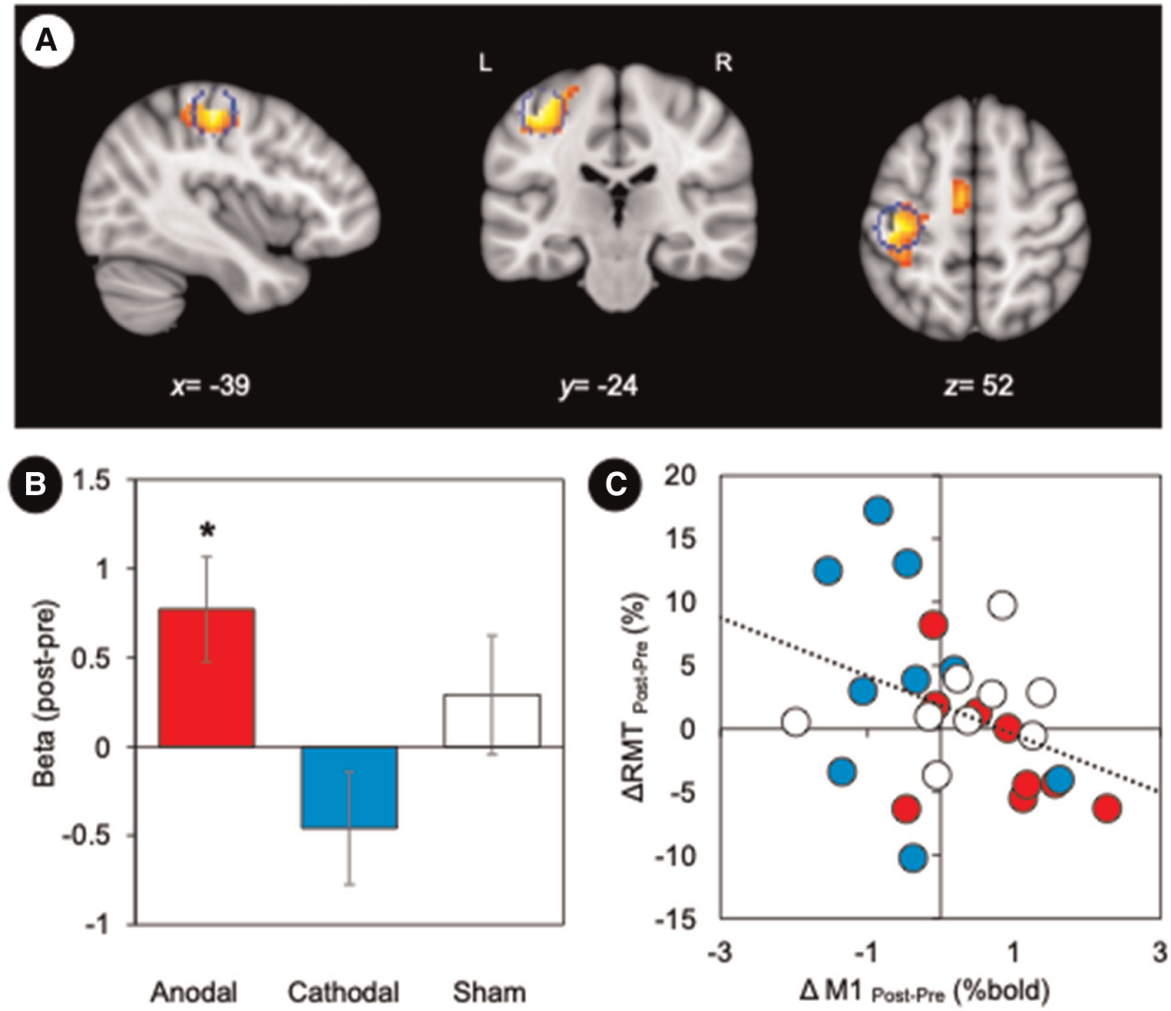
Motor cortex plasticity. (**A**) M1 location defined by the maximally activated voxel in the contralateral (left) hemisphere. Statistical maps based on the conjunction map of the responses elicited by the hand motor task (pre-stimulation). The coordinates of the group maxima are reported on the structural images from the MNI template, and a sphere of 6 mm radius marked for reference. Coordinates of the group maxima of activation are reported in Table 1. All images are displayed with a statistical significance of *p* < 0.005 at the voxel level and *p* < 0.05 (ks > 30) at a cluster level with false-discovery rate (FDR) correction. (**B**) GLM parameter estimates (weighted beta values) representing the magnitude of M1 responses to tDCS stimulation. Error bars represent standard error. *p < 0.05. (**C**) Relationship between changes in RMT and M1 activity. We found a significant negative correlation between RMT changes (post—pre) and M1 activity changes (post—pre) (*r* = −0.40, *p* = 0.038).

To assess the functional coupling to the site where non-invasive brain stimulation was effective at inducing neuroplasticity, a seed region was defined in the left M1 (target region, MNI: −39, −24, 54) based on the GLM task results (Figure 4A). Subsequent analyses of the regional activity changes (post—pre) were conducted to examine the effect of stimulation (Figure 4B). We found that anodal stimulation significantly increased regional activity in the left M1 (one-sample Wilcoxon Signed Rank Test, *p* = 0.025) whereas cathodal stimulation decreased activity (*p* = 0.07). We also assessed the stimulation effect on RMT by performing a correlation analysis between RMT changes (post—pre) and M1 activity changes (post—pre) (Figure 4C). We found a significant negative correlation (*r* = −0.40, *p* = 0.038). Individuals with increased RMT after stimulation showed decreased regional activity in the left M1 (target region).

### Whole-brain gPPI connectivity results

**Figure 5:**
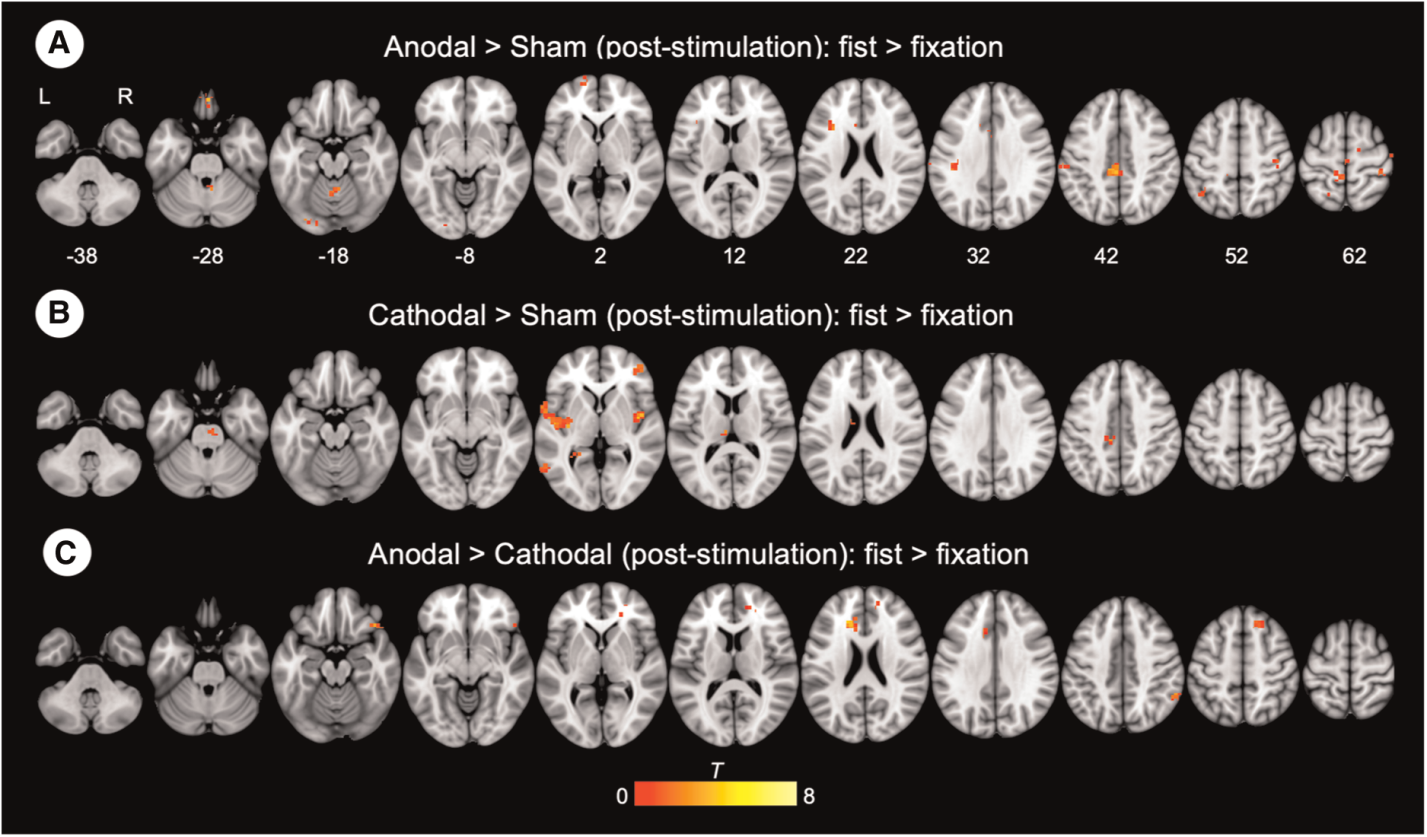
Whole-brain gPPI results for the left M1 during hand motor task. (**A, B, C**) Generalized psychophysiological interaction (gPPI) analysis: interaction of stimulation type x task condition (fist > fixation) on M1 connectivity. R (right) and L (left) indicate image orientations for slices in axial planes. Reference positions are in MNI coordinates. All images are displayed with a statistical significance of *p* < 0.005 at the voxel level and *p* < 0.05 (ks > 30) at a cluster level with false-discovery rate (FDR) correction.

Connectivity changes during the hand motor task were estimated with gPPI. This allowed us to directly contrast functional connectivity during fist clenching with functional connectivity in the fixation (rest) condition. The gPPI analysis with the left M1 as a seed region revealed that anodal stimulation enhanced connectivity with middle/posterior cingulate cortex relative to sham stimulation (Figure 5A), whereas cathodal stimulation strengthened connectivity with insula and operculum (Figure 5B). The anodal stimulation significantly increased connectivity between the M1 and anterior and middle cingulate cortex compared to the cathodal stimulation (Figure 5C). These results are summarized in Table 2.

**Table 2:** The results of whole brain analysis of gPPI.

Contrast	Regions	Cluster size	*Z*	MNI coordinate		
				x	y	z
Anodal > Sham						
Post-session	Posterior cingulate cortex	206	3.51	−2	−34	42
fist > fixation			3.39	−10	−30	42
	Middle cingulate cortex		3.14	−2	−26	38
Cathodal > Sham						
Post-session	Insula	505	3.75	−36	−12	6
fist > fixation	Central Operculum		3.6	−48	−12	2
			3.46	−56	2	0
Anodal > Cathodal						
Post-session	Anterior cingulate cortex	264	3.51	−14	26	18
			3.39	−12	32	24
	Middle cingulate cortex		3.14	−12	18	30

The left M1 was used as the seed region.

## Discussion

Neuromodulation with tDCS is an emerging tool for the treatment of chronic pain. However, issues relating to poor outcomes and replicability have compromised this method (43). We considered the concept that a target regions connectivity profile is fundamental to the therapeutic effects of modulation (44). We combined tDCS and fMRI to measure changes in task-specific activity and interregional functional connectivity between the primary motor cortex (M1) and the whole brain. In the following discussion, we consider the potential mechanisms underlying the observed neuroplastic after-effects on excitability induced by tDCS, and its potential relevance for the therapeutic effects of modulating disease-specific symptoms in chronic pain.

### Modulation of task-dependent activity

tDCS has been shown to induce long-lasting and polarity-specific changes in motor cortex excitability (13, 45, 46) that relates to modifications in synaptic efficacy and LTP/LDP-like plasticity (47, 48). We demonstrated that tDCS applied to M1 (the target region) induces task-specific facilitation of local brain activity after anodal tDCS, with the stimulation effects having a negative relationship to the RMT. Previous studies have shown that functional changes in movement-related activity are modulated by tDCS (31, 49, 50). For example, Stagg et al. (31) reported a significant increase of M1 activation after anodal tDCS, which is in agreement with the findings of this study. However, in our study, the increases in brain activity were more widespread and extended to include several regions that potentially belong to the same network, such as the insular and cingulate cortex. Although widespread cortical changes have been reported as a result of tDCS (31, 49, 50), it is still not clear to what extent M1 stimulation modulates task-related activity outside of the motor system. This is in part due to the experimental differences between studies, including the tDCS protocols, fMRI acquisition parameters, and the specifics of the task. However, to our knowledge this is the first study demonstrating the modulating effects of M1-tDCS on a functionally distinct set of brain regions that may contribute to a variety of complex functions relevant for targeting pain (51, 52).

### Modulation of task-dependent functional connectivity

The versatility of fMRI affords the additional opportunity to understand how brain regions interact in a task-dependent manner. Context-dependent connectivity, or the connectivity during the hand motor task conditions (i.e., fist/fixation), was examined using the left M1 as a seed region and revealed that anodal stimulation enhanced connectivity with middle/posterior cingulate cortex relative to sham stimulation, whereas cathodal stimulation strengthened connectivity with the insula and operculum. A further comparison of the two active tDCS groups showed that the anterior and midcingulate cortex is most susceptible to the polarity-dependent effects of M1 stimulation. Previous studies have exclusively focused on the effects of tDCS-induced functional connectivity alterations independent of task-related activity and performance (25, 26, 32, 33). However, to date, there have been no studies examining the psychophysiological interactions of motor cortex tDCS on such networks. The remote impact of tDCS is consistent with previous work that used TMS to modulate effective connectivity between M1 and other remote brain areas relevant for pain processing and modulation (53). Based on these data, we speculate that both tDCS and TMS can extensively modulate distributed functional systems in the human brain (51, 52), and that the spreading effects are not confined to a single network of interest. It may be the case that functional activity and connectivity of other areas are related to the plasticity-inducing effects of M1 stimulation, albeit to a weaker degree than the significant findings in the present study.

### Conclusion and future perspectives

tDCS of the primary motor cortex is promising treatment for several brain disorders including chronic, but its mechanism of action remains unclear (Gan et al., 2021). The findings presented here support the hypothesis that a target regions connectivity profile is relevant to the therapeutic effects of modulation (44). The downstream regions susceptible to tDCS manipulation include the operculo-insular and cingulate cortex, which have reciprocal interconnections relevant for supporting complex behavioral functions, such as pain, valence, and arousal. No measures of corticospinal excitability (i.e., resting motor threshold, RMT) could independently explain the observed changes in brain function. However, as with any exploratory investigation, there several limitations we must consider. The main issue of this study pertains to the small cohort of subjects used within the stimulation groups (54). The next step will be to repeat this type of investigation with a much larger cohort of subjects and select patient groups, especially those with pain conditions relevant for tDCS therapy. The lack of an explicit nociceptive stimulus or task means that caution must be applied when interpreting the selected brain areas as pain specific. Nevertheless, these results offer opportunities to improve outcomes of tDCS for the individual patient based on the degree of presumed neuroplasticity. Further research is still warranted to address the optimal stimulation target and parameters for those with disease-specific symptoms of chronic pain.

## Data Availability

The raw data supporting the conclusions of this article will be available on request to the corresponding author.
